# Obituary for Herbert Gottweis, Professor of Political Science, University of Vienna: Born 8 February 1958 in Vienna, died 31 March 2014 in Vienna

**DOI:** 10.1186/s40504-014-0012-9

**Published:** 2014-04-30

**Authors:** Barbara Prainsack

**Affiliations:** King’s College London, London, UK

On 31 March 2014, Herbert Gottweis died in Vienna, aged 56. His death is a big loss to his family and friends, and to the international scholarly community.

Herbert studied Political Science at the University of Vienna, where he also obtained his doctoral degree in 1984. He became an Assistant Professor at the University of Salzburg a year later. During his tenure in Salzburg he also held various research and teaching fellowships at some of the world’s best universities: He was an Erwin Schrödinger Fellow at the Center for European Studies at Harvard University (1989/90), an Andrew Mellon Foundation Fellow at MIT (1992/93), an Assistant Professor at the Department of Science and Technology Studies at Cornell University (1993/1995), and a Visiting Professor at the Department of Social Studies at Hong Kong University of Science and Technology. The heavy travelling schedule that he maintained early in his academic career remained with him until the end of his life: Herbert embodied both deep-rootedness and openness for adventure. This combination also applied to his intellectual work: while he maintained a strong commitment to political science (‘Where is the power?’, he often asked colleagues when he felt their work lacked focus on what really matters), he also had an almost uncanny ability to spot issues in the domain of science and technology that would become politically pertinent or controversial, and he stretched the boundaries of his home discipline to address these.

His habilitation project, on which he embarked in the 1990s, was highly unusual and innovative within political science at that time: Using a discursive analytic approach, Herbert explored how genetic engineering had become a controversial technology. This habilitation thesis was published with MIT Press two years later (*Governing Molecules: Discursive Politics of Genetic Engineering in Europe and the United States*, 1998) and it remains a major work in the field of critical studies of biotechnology regulation and Science and Technology Studies (STS).

Herbert accepted a Professorship at the Department of Political Science at the University of Vienna in 1998, a position which he retained until his death. I was finishing my Master’s degree at the Department when he arrived. I could not wait to take a seminar with the new Professor who had just joined us from Salzburg, and who was the subject of a lot of talk among students: He taught courses that most of us had not considered possible in a political science curriculum, ranging from statehood and collective identities to the governance of biotechnology. Also his teaching style was rather unusual: He required us to engage with the assigned readings – which were always well considered and stimulating – but he welcomed members of the group to draw upon any other materials that we felt were relevant. Our group did not always agree on what others considered ‘relevant’, and we had heated arguments in almost every seminar session. But I cannot recall any other course where students worked so hard, not because we were concerned about the mark, but because he created an intellectual environment that made us want to excel. The atmosphere that I remember from these days is that Herbert made us feel that everything was possible.

Students and researchers flocked to Herbert, and his international reputation continued to grow as well. In the following years, he continued to be an active scholar and institution builder, both nationally and internationally. At the Department of Political Science in Vienna, he founded the *Life Science Governance Platform*, which helped to further establish and later consolidate the innovative work at the interface of science, society and governance that Herbert stood for within the Austrian political science community. The *Life Science Governance Platform* has been the home for dozens of young researchers, and temporary home for many visiting fellows from various disciplines from all over the world. From 2005 Herbert also served as Vice President of the Austrian Science Foundation, making an invaluable contribution to the Austrian scientific community as a whole.

Internationally, Herbert’s work has had a significant impact in two main areas: He enhanced the quality and visibility of governance studies within the field of STS, and he facilitated the engagement of political scientists with STS and vice versa. He achieved this not only through his scholarship, but also through his active and practical support of groups and initiatives at the interface of life science and governance: For example, he played an important role in shaping the Centre for Society and the Life Sciences (CSG) in Nijmegen, NL, as a member of their Scientific Advisory Board, and he was an active member of several professional bodies and editorial boards, including the that of the LSSP.

Herbert was also a key figure in the establishment of the field of interpretive policy studies in Europe. In 2003 he established, with Hendrik Wagenaar, a ‘Standing Group’ on interpretive policy analysis within the European Consortium of Political Research (ECPR), thereby obtaining recognition for interpretive approaches within the international political science establishment. He was also involved, from the very start in 2005, with the annual conference of Interpretive Policy Analysis (organising the 2013 edition in Vienna), which has become one of the major platforms for young and senior scholars in this burgeoning field. He also served in the Editorial Council of the journal *Critical Policy Studies*.

Although he had lots of friends all over the world, Herbert was a private person. But what was very apparent to everybody who knew him was how deeply devoted he was to his family. He loved his family with all his heart. He was also a very active mentor and supporter of his students and researchers. Countless people who had worked or studied with Herbert abroad also told me over the years how he had helped them in their thinking, their career, and how much they valued his friendship.

In many ways Herbert was as passionate about the careers of his students and younger colleagues as he was about his own; he urged us to publish from the earliest stages of our projects, organised scholarships and visiting fellowships for us at institutions all over the world, and was a master in writing grant proposals - from which many of us benefitted both directly and indirectly. But perhaps the most valuable thing that we could learn from Herbert was a firm trust in our own research instincts. Herbert went against the mainstream in much of his work, despite doubts that others raised about such a risky strategy. During the time that I worked with Herbert–first as a doctoral student, then as a post-doc, research associate, and lecturer–that confidence had rubbed off on me, in ways that were perhaps unintended by him: I went against his advice on several occasions, sometimes regretting later that I had not listened to him, and sometimes finding that I had done the right thing. He never held it against us when we did our own thing, even against his own advice. He let us steer our own course and supported us nevertheless. I only discovered the full value of this later, long after I had left Vienna.

Herbert also was very helpful in the context of my move to the UK, and other colleagues have had similar experiences: it gave him great joy to know that we succeeded elsewhere. He was great at attracting people to his group, but he was also good at letting them go. This is one of the many reasons for why it is so difficult for many of us to let him go.

Herbert Gottweis is survived by his wife Ursula and his children Clara, Raphael and Theresa. He is painfully missed also by his friends, colleagues and students from all over the world. His work and life will continue to influence and inspire us.

Barbara Prainsack

Professor of Sociology

Department of Social Science, Health & Medicine

King’s College London

